# Supplementary material for: Cognition and motor function: The gait and cognition pooled index
Source: PLoS One. 2020 Sep 11;15(9):e0238690. doi: 10.1371/journal.pone.0238690 (PMC7485843; doi:10.1371/journal.pone.0238690)
Supplement: S1 Appendix — (DOCX) [file pone.0238690.s001.docx]

**S1 Appendix: ADAS-Cog-Proxy Development Details**

Section A

ADAS-Cog-Proxy development was performed in five steps, which are summarized in Figure A and described below.

**ADNI Development Subset (70%)**

**n=401 (144 NC, 257 MCI)**

Build candidate ADAS-Cog-Proxy models.

*Model type [covariates], preliminary accuracy*

M1: LINEAR [MMSE], 56.4%

M2: GAM [MMSE], 57.6%

M3: GAM [MMSE, RAVLT], 63.6%

M4: GAM [MMSE, RAVLT, CDR-SB], 65.6%

M5: GAM [MMSE, RAVLT, CDR-SB, DSfwd, DSbkwd, TMTA, TMTB], 68.4%

**ADNI**

**n=573 (206 NC, 367 MCI)**

Build final ADAS-Cog-Proxy model.

**GABS**

**n=109 (12 NC, 19 SCI, 78 MCI)**

Use MICE to obtain missing model covariate values. Model applied to 5 imputed datasets, and the average of estimates taken as final ADAS-Cog-Proxy score.

**ADNI**

**n=573 (206 NC, 367 MCI)**

2

1

3

**ADNI Testing Subset (30%)**

**n=172 (62 NC, 110 MCI)**

Estimate accuracy of best candidate model (M4).

68.6% scores predicted within 3 points,

88.4% within 5 points.

rho=0.70 (P<0.001)

5

4

**Figure A. ADAS-Cog-Proxy model development and application.** *Note.* Grey boxes indicate steps. *Abbreviations:* ADAS-Cog=Alzheimer’s Disease Assessment Scale-Cognitive Subscale, ADNI=Alzheimer’s Disease Neuroimaging Initiative Phase 1 Study, CDR-SB=Clinical Dementia Rating Scale-Sum of Boxes, DSfwd=Digit Span Forward, DSbkwd=DS Backward, GAM=Generalized Additive Models, GABS=Gait and Brain Study, MCI=Mild Cognitive Impairment, NC=Normal Cognition, RAVLT=Rey Auditory Verbal Learning Test, TMT=Trail Making Test, SCI=Subjective Cognitive Impairment.

**Step 1:** Participants in the Alzheimer’s Disease Neuroimaging Initiative Phase 1 Study (ADNI1) with Normal Cognition (NC) or Mild Cognitive Impairment (MCI) were randomly divided into 70% development (n=401) and 30% (n=172) testing subsets.

**Step 2:** Five candidate models were constructed in the development subset, including one linear model and four generalized additive models (GAM). Candidate covariates (seven cognitive tests available in both ADNI1 and GABS) were added to subsequent GAMs in order of theoretical similarity to questions and measurement domains of the Alzheimer’s Disease Assessment Scale-Cognitive Subscale (ADAS-Cog). For each candidate model, preliminary accuracy was assessed as the percentage of participants with predicted ADAS-Cog scores within three points of their observed score.

**Step 3**: Accuracy of the best candidate model (M4) was estimated in the testing subset. M4 was selected as the best model based on preliminary accuracy and covariates: the accuracy of M4 was only slightly worse than M5 while the covariates of M4 were fewer and more theoretically sound than those in M5 meaning the M4 covariates measure similar domains to the ADAS-Cog. Selecting a simpler model with stronger theoretical grounds is expected to reduce overfitting and produce more accurate estimates when used with a new dataset (i.e. Gait and Brain Study (GABS)).

**Step 4:** Spearman’s rank correlation (rho) between predicted and observed scores was also calculated. Development and testing subsets were recombined to build the final GAM. To assess similarity of participants in ADNI1 and GABS, ADAS-Cog-Proxy GAM covariates were compared between the two datasets (Table A). As a final check for ADAS-Cog-Proxy GAM performance, the accuracy of a previously published univariate linear model for converting between Mini-Mental State Examination (MMSE) and ADAS-Cog scores was assessed (1). This model was developed in a sample of older adults with MCI and Alzheimer’s Disease, which indicates higher levels of cognitive dysfunction than the ADNI1 or GABS sample, suggesting the model may not perform well enough for the purpose of approximating ADAS-Cog scores in GABS. Indeed, this model predicted 53.2% of ADAS-Cog scores within three points and 76.1% within five points of observed ADAS-Cog scores on combined development and testing ADNI1 data.

**Step 5:** Multivariate Imputation by Chained Equations (MICE) was used to impute missing GAM covariates GABS (Section B), and ADAS-Cog-Proxy GAM scores were estimated for all participants.

**Table A: Range of ADAS-Cog-Proxy GAM Covariate Scores in ADNI1 and GABS**

| **Covariate**  **Range (min, max)** | **MMSE**  **0, 30** | **RAVLT**  **0, 45** | **CDR-SB**  **0, 4.5** |
| --- | --- | --- | --- |
| ADNI data used to build ADAS-Cog-Proxy model | | | |
| Observed Range | 23, 30 | 5, 38 | 0, 4.5 |
| GABS Baseline | | | |
| Observed Range | 18, 30 | 8, 33 | 0, 4 |
| n below/above | 7/0 | 0 | 0 |
| Total n out of range | 7 | 0 | 0 |
| GABS 6-month follow-up | | | |
| Observed Range | 21, 30 | 5, 34 | 0, 4 |
| n below/above | 3/0 | 0 | 0/0 |
| Total n out of range | 3 | 0 | 0 |
| GABS 12-month follow-up | | | |
| Observed Range | 20, 30 | 6, 39 | 0, 2.5 |
| n below/above | 2 | 0/1 | 0/0 |
| Total n out of range | 2 | 1 | 0 |
| GABS 24-month follow-up | | | |
| Observed Range | 21, 30 | 9, 43 | 0.5, 5 |
| n below/above | 2/0 | 0/1 | 0/1 |
| Total n out of range | 2 | 1 | 1 |
| GABS 36-month follow-up | | | |
| Observed Range | 20, 30 | 6, 38 | 0.5, 3.5 |
| n below/above | 1/0 | 0 | 0/0 |
| Total n out of range | 1 | 0 | 0 |
| GABS 48-month follow-up | | | |
| Observed Range | 22, 30 | 5, 38 | 0.5, 4.0 |
| n below/above | 1/0 | 0 | 0/0 |
| Total n out of range | 1 | 0 | 0 |

Section B: Missing data

**Table B. Missing ADAS-Cog-Proxy Model Covariates in the Gait and Brain Study.**

| **Timepoint**  **(months)** | **n** | **MMSE** | **RAVLT** | **CDR-SB Total (no collaborator)** | **CDR-SB & RAVLT** |
| --- | --- | --- | --- | --- | --- |
| 0 | 109 | 0 | 29 | 68 (53) | 24 |
| 6 | 86 | 0 | 28 | 63 (50) | 25 |
| 12 | 73 | 0 | 16 | 57 (40) | 15 |
| 24 | 55 | 0 | 3 | 39 (33) | 3 |
| 36 | 35 | 0 | 0 | 21 (21) | 0 |
| 48 | 24 | 0 | 0 | 20 (19) | 0 |

*Note.* Multiple imputation by chained equations was used to impute missing values, using the imputation method of predictive mean matching. Abbreviations: MMSE=Mini-Menta

l State Examination, RAVLT=Rey Auditory Verbal Learning Test (3 trials), CDR-SB=Clinical Dementia Rating Scale-Sum of Boxes, m=months. Missingness of the CDR-SB is high largely due to participants not attending study visits with a suitable partner to assist with CDR-SB assessment.

In accordance with published guidelines (2-4), predictor matrices included all GAM covariates, predictors of the outcome ADAS-Cog scores, variables that include a lot of variance as roughly identified by correlation with the target variables to be imputed, and no variables that had a lot of missing values within the subgroup of people with missing RAVLT and CDR-SB scores. It has also been suggested to include variables related to non-response. The main reason the CDR-SB scores are missing is if no collaborator was present to report on behalf of the patient; however, there was not a variable in the dataset expected to provide indication of this. MICE was not performed on the entire GABS database due to multicollinearity and computational restrictions and because research suggests that there is little improvement in accuracy when imputation considers more than 15-25 predictors.

The final list of included covariates for MICE was: Baseline Diagnosis, MMSE, MoCA, MoCAMIS, MoCAEIS, MoCAVIS, MoCALIS, MoCAAIS, MoCAOIS, CDR, Trail A, Trail B, Digit Forward, Digit Backward, Letter Number, RAVLT, BNT, FAB, number of falls in past 6 months, IADL, RSEO (balance), Gait Velocity, and Gait Velocity while counting backwards by ones, from the time point of interest, as well as CDR and RAVLT scores from the previous visit (T6 to T48 visit imputations) or a future visit (baseline visit imputations). The MICE procedure was performed separately for each time point to allow for the exclusion of observations that were missing simply because the corresponding participants did not have the follow-up visit.

**References:**

1. Solomon TM, DeBros GB, Budson AE, Mirkovic N, Murphy CA, Solomon PR. Correlational analysis of 5 commonly used measures of cognitive functioning and mental status: an update. *Am J Alzheimers Dis Other Demen*. 2014;29(8):718-722. doi:10.1177/1533317514534761
2. van Buuren S, Groothuis-Oudshoorn K. mice: Multivariate imputation by chained equations in R. *J Stat Softw*. 2011;45(3). doi:10.18637/jss.v045.i03.
3. van Buuren S, Oudshoorn K. *Flexible Multivariate Imputation by MICE*. Netherlands Organization for Applied Science Research;1999.
4. White IR, Royston P, Wood AM. Multiple imputation using chained equations: issues and guidance for practice. *Stat Med*. 2011;30(4):377-399. doi:10.1002/sim.4067.
