# Supplementary material for: Cognition and motor function: The gait and cognition pooled index
Source: PLoS One. 2020 Sep 11;15(9):e0238690. doi: 10.1371/journal.pone.0238690 (PMC7485843; doi:10.1371/journal.pone.0238690)
Supplement: S1 Table — (DOCX) [file pone.0238690.s002.docx]

**S1 Table: Secondary Analysis: Mini Mental State Examination responsiveness to group-level within-person measured change over time.**

| **Sample size** | **Months of follow-up** | **Standardized Response Mean**  **(95% bootstrap confidence interval)** |
| --- | --- | --- |
| 86 | 6 | 0.17  (-0.04, 0.41) |
| 72 | 12 | 0.27  (0.03, 0.54) |
| 55 | 24 | 0.18  (-0.09, 0.44) |
| 35 | 36 | 0.46  (0.20, 0.77) |
| 24 | 48 | 1.35  (0.82, 2.44) |

*Note:* one person had a missing MMSE score for 12 months of follow up and was excluded. All other sample sizes are the same as Table 3 in the main text.
