# Supplementary material for: Cognition and motor function: The gait and cognition pooled index
Source: PLoS One. 2020 Sep 11;15(9):e0238690. doi: 10.1371/journal.pone.0238690 (PMC7485843; doi:10.1371/journal.pone.0238690)
Supplement: Attachment — Submitted filename: Response to Reviewers.docx [file pone.0238690.s004.docx]

Reviewer #4: The introduction and discussion of this paper are substantially improved. Recommended changes:

Abstract:
Line 36/37: incomplete sentence. Please revise.

**Response: We have revised this sentence to: *Final selected variables for the Pooled Index include gait velocity, dual-task cost of gait velocity, and an ADAS-Cog-Proxy (statistical approximation of the ADAS-Cog using similar cognitive tests).***

Introduction:
Line 52: I disagree with the use of term “emerging research." Instead, “multiple studies have reported relationships between cognitive and motor function in pre-dementia syndromes". Citations are needed.

Here are just a few studies that could be cited:
Aggarwal NT, Wilson RS, Beck TL, Bienias JL, Bennett DA. Motor dysfunction in mild cognitive impairment and the risk of incident Alzheimer disease. Arch Neurol 2006;63:1763-9.

Mielke MM, Roberts RO, Savica R, et al. Assessing the Temporal Relationship Between Cognition and Gait: Slow Gait Predicts Cognitive Decline in the Mayo Clinic Study of Aging. J Gerontol A Biol Sci Med Sci 2013; 68:929-37.

Verghese J, Robbins M, Holtzer R, et al. Gait dysfunction in mild cognitive impairment syndromes. J Am Geriatr Soc 2008;56:1244-51

Buchman AS, Bennett DA. Loss of motor function in preclinical Alzheimer's disease. Expert Rev Neurother 2011;11:665-76.

Liu-Ambrose TY, Ashe MC, Graf P, et al. Increased risk of falling in older community-dwelling women with mild
cognitive impairment. Phys Ther. 2008; 88:1482–1491.

Waite LM, Grayson DA, Piguet O, et al. Gait slowing as a predictor of incident dementia: 6-year longitudinal data from the Sydney Older Persons Study. J Neurol Sci. 2005;229–230:89–93.

**Response: *We agree with the reviewer’s idea and are thankful for the suggested citations; these have been incorporated.***

Line 53-55: The sentences "There is a need……” is vague. I recommend replacing with a clear and accessible statement.

**Response: *We revised the sentence to read, “There is a need for outcome measures that reflect these advancements and are more responsive for present research settings, while maintaining compatibility with historical measurement techniques.”***

Line 56: Mild Cognitive Impairment - No need to capitalize

**Response: *we removed the capitalization.***

Line 65: ….Natural history. add “of disease progression” or “of MCI” (Natural history of disease progression)

**Response: we added *‘of disease progression’.***

Line 87-88 Unclear sentence - “Our literature review found…

**Response: *The sentence has been revised to: Our literature review of modifications made to the ADAS-Cog since its development did not find any revisions whereby motor function or DTC assessments were added to the ADAS-Cog***

Line 91-93 Remove this statement: “ While we focus on the ADAS-Cog given its prominence and strong history in the field, but our methodological approach can serve as an example for researchers to follow for other contexts and measures as well.”

***Response: We removed the sentence.***
